# Traffic Flow Prediction Model Based on the Combination of Improved Gated Recurrent Unit and Graph Convolutional Network

**DOI:** 10.3389/fbioe.2022.804454

**Published:** 2022-02-14

**Authors:** Yun Zhao, Xue Han, Xing Xu

**Affiliations:** ^1^ School of Information and Electronic Engineering, Zhejiang University of Science and Technology, Hangzhou, China; ^2^ Mechanical and Energy Engineering, Zhejiang University of Science and Technology, Hangzhou, China

**Keywords:** traffic flow prediction, deep learning, graph convolutional network, gated recurrent unit, temporal and spatial correlations

## Abstract

With the rapid economic growth and the continuous increase in population, cars have become a necessity for most people to travel. The increase in the number of cars is accompanied by serious traffic congestion. In order to alleviate traffic congestion, many places have introduced policies such as vehicle restriction, and intelligent transportation systems have gradually been put into use. Due to the chaotic complexity of the traffic road network and the short-term mobility of the population, traffic flow prediction is affected by many complex factors, and an effective traffic flow forecasting system is very challenging. This paper proposes a model to predict the traffic flow of Wenyi Road in Hangzhou. Wenyi Road consists of four crossroads. The four intersections have the same changing trend in traffic flow at the same time, which indicates that the roads influence each other spatially, and the traffic flow has spatial and temporal correlation. Based on this feature of traffic flow, we propose the IMgru model to better extract the traffic flow temporal characteristics. In addition, the IMgruGcn model is proposed, which combines the graph convolutional network (GCN) module and the IMgru module, to extract the spatiotemporal features of traffic flow simultaneously. Finally, according to the morning and evening peak characteristics of Hangzhou, the Wenyi Road dataset is divided into peak period and off-peak period for prediction. Comparing the IMgruGcn model with five baseline models and a state-of-the-art method, the IMgruGcn model achieves better results. Best results were also achieved on a public dataset, demonstrating the generalization ability of the IMgruGcn model.

## Introduction

The number of motor vehicles is rising with the rapid development of technology and economy and caused more serious traffic congestion. How to relieve traffic congestion effectively has become a hot topic of concern (Hou et al., 2019). In order to relieve traffic pressure and improve the smoothness of travel, various solutions have emerged, such as increasing road width, traffic flow limiting according to single and double license plates, using public transportation, and developing traffic management systems.

The main strategy to solve traffic congestion in the early days was to enhance road construction and increase the traffic capacity of the roads to meet the traffic demand. Since the rate of road construction was far from keeping up with the increase in vehicles and the limitation in urban area (Yu et al., 2019), this led to severe traffic jams during the peak hours of travel to work. The research focus has shifted to how to make the best use of urban roads by improving the utilization of existing roads. Intelligent transportation systems (ITS) have also emerged (Do et al., 2019), to manage vehicles intelligently and direct traffic flow, to change the spatial and temporal distribution of vehicles in the road network and equalize traffic flow.

Early traffic flow detection mainly relied on manual survey records, and with the development of computer and electronic information technology, detection techniques were gradually improved. Domestic and foreign researchers proposed automatic acquisition methods, such as parameter threshold method, pattern recognition method, and numerical analysis method. The latter turned to the use of GIS technology to locate and collect data such as vehicle travel time and speed, with travel time as the main metric parameter. However, the lack of proper understanding and grasp of the mechanism of urban road congestion and spatiotemporal characteristics of traffic flow led to the recurrence of traffic congestion, and sudden traffic accidents has caused episodic traffic congestion problems. It cannot make a correct early warning of the place, time, radius of the traffic congestions, and duration of occurrence. Therefore, the existing strategies on controlling traffic congestion also tend to stay at the level of theoretical analysis, lacking systematic and real-time operational measures, which require intelligent traffic control and guidance.

**FIGURE 1:**
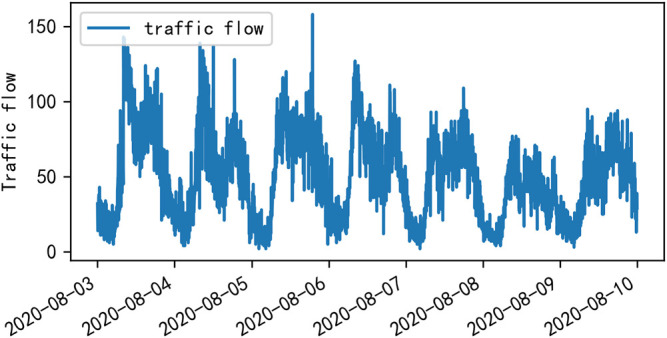
Visualization of traffic flow in 1 week at the Yile intersection of Wenyi Road.

**FIGURE 2:**
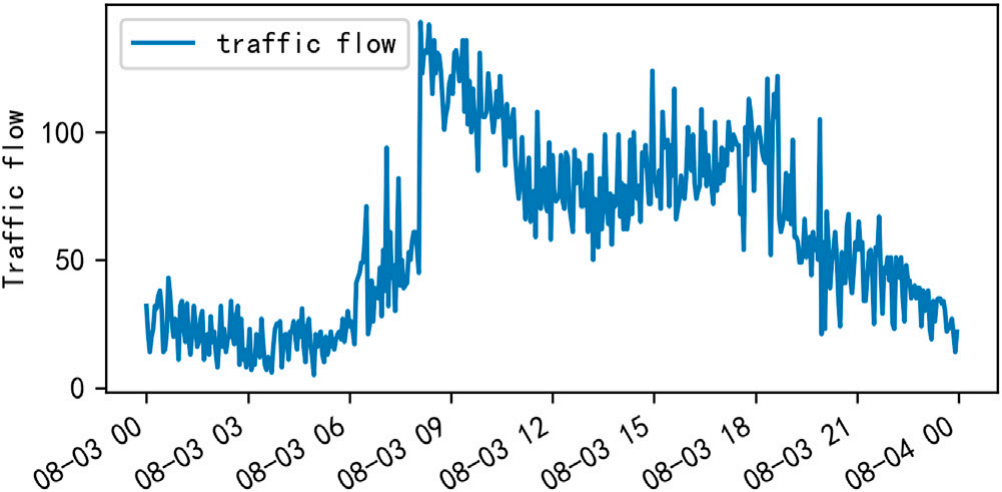
Visualization of traffic flow in 1 day at the Yile intersection of Wenyi Road.

Traffic control and guidance system are the main direction of ITS research, and the main problem of traffic control and guidance is traffic flow prediction (Tang et al., 2013). Urban roads are composed of intricate road sections and various intersections. The composition of traffic flow varies from time to time and place to place. The causes of traffic congestion, the location of occurrence, and the radius of the traffic congestions of influence are also different, and traffic flow shows obvious characteristics of temporal and spatial distribution. Figure 1 shows the obvious cyclical changes of traffic flow during a week. Figure 2 shows the variation of traffic flow during a day, and it can be seen that there is a peak period in the morning and one in the afternoon.

Traffic flow prediction is one of the ways of data collection and processing in intelligent transportation systems. How to use effective traffic information to predict the traffic flow conditions in the next few minutes or hours so that drivers can better choose smooth roads and, thus, effectively reduce traffic congestion is one method called short-time traffic flow prediction method (Liu and Guan, 2004). Short-time traffic flow prediction methods are divided into three main categories (Luo et al., 2019): prediction models based on linear statistical theory, prediction models based on nonlinear theory, and prediction models based on artificial intelligence theory. There are also some hybrid prediction models (Guan and Chen, 2006), such as wavelet analysis models combined with ARIMA models (Dou et al., 2009), in order to give full play to the advantages of each prediction model, while having the characteristics of integration and complementarity between models so that the road traffic flow can be predicted accurately and comprehensively, and the prediction accuracy can be improved effectively.

With the continuous development of the artificial intelligence theory, traffic flow prediction has also started to adopt deep learning methods. The essence of deep learning is artificial neural network. Neural network is a mathematical or computational model that mimics the structure and function of a biological neural network (the central nervous system of animals, especially the brain) for estimating or approximating a function. The neural network consists of a large number of artificial neurons linked for computation and is capable of simple decision-making abilities and simple judgments similar to those in humans.

Domestic and foreign scholars have used deep learning as the focus of their research (Yi et al., 2017) and achieved certain results. Huang et al. (2014) proposed a deep architecture model, which consisted of a deep belief network (DBN) at the bottom and a multi-task regression layer at the top, to predict traffic flow. Yi et al. (2019) studied the implementation of a deep long short-term memory recurrent neural network (LSTM-RNN) in a highway system. These methods confirmed the effectiveness and feasibility of deep learning in the direction of traffic flow prediction, but did not fully consider the spatiotemporal characteristics of traffic flow. Wu et al. (2018) proposed a DNN-based traffic flow prediction model (DNN-BTF) to improve the prediction accuracy, using convolutional neural networks to mine spatial features and recurrent neural networks to mine temporal features of traffic flow. Wang and Xu proposed an LSTM-RNN-based time series prediction model for urban highway traffic flow in a deep learning framework, which reconstructed the traffic time series by the integrated spatiotemporal correlation of traffic flow, so that the LSTM-RNN gains and enhances data mining capability. Zhene et al. (2018) proposed a deep learning model based on CNN and RNN, using matrix traffic as input, extracting traffic features using CNN, predicting feature evolution using RNN, and mixing the two models to achieve traffic flow prediction. Although these methods considered spatiotemporal correlation, they did not address the long-term memory problem and the gradient problem in backpropagation.

This paper studied the prediction of traffic flow from both spatial and temporal aspects, combining the temporal feature extraction module and the spatial feature extraction module. First, we obtained the spatial characteristics of traffic flow by graph convolutional network and then predicted the future traffic flow based on the spatiotemporal correlation of traffic flow. The IM module was also proposed in the temporal feature extraction module, to enhance the connection of traffic flow between input and hidden state, and improve the traffic flow prediction capability.

When extracting temporal features of traffic flow, it is necessary to predict the next moment by remembering the information of the previous moment, but it is difficult to remember the input information that is too far apart. Therefore, the problem of long time dependence needs to be solved. The GRU model is used to obtain the temporal features of traffic flow because it has a simpler structure and less computation, which can reduce the risk of overfitting. For the traditional GRU model, the inputs and the hidden states passed down from the previous moment are independent of each other until they enter the model interior. They only interact with the information inside the GRU. This may lead to the loss of valid information. We proposed the IMgru model with a richer interactive representation of the inputs and hidden states, which enhances the significant information, reduces the secondary information, and enhances the modeling capability of the model.

In addition, since the traffic flow at the four crossroads of Wenyi Road interact with each other spatially. It is not accurate to predict traffic flow only by the temporal characteristics of traffic flow. Therefore, a combination of spatial and temporal characteristics is needed to predict the traffic flow.

In this paper, the traffic flow dataset of Wenyi Road in Hangzhou was collected, and the prediction effect was affected by the dense flow of people and vehicles during the peak hours of weekdays in Hangzhou, which was very prone to traffic accidents and was affected by weather and other factors. Therefore, the Wenyi Road dataset was divided into peak period and off-peak period for separate prediction. In order to achieve better results in the off-peak period, the model proposed in this paper was applied to Wenyi Road dataset and two public datasets. The experiments showed that the IMgruGcn model can mine the spatiotemporal correlation of traffic flow, and the prediction results are better than other models.

## Related work

Traditional traffic flow forecasting methods are mainly based on linear statistical models and nonlinear theory-based models. Linear statistical-based models use mathematical statistical theory to analyze historical traffic and predict future traffic, including autoregressive sliding average (ARIMA) models (Williams et al., 1998), historical average (HA) models (Chang et al., 2011), Kalman filter prediction models (Kalman and R, 1960), and support vector regression classifier (SVR) models (Smola and Scholkopf, 2004). The historical averaging model is a simple method and can solve the problem of traffic flow variations at different times to some extent, but its prediction is static and cannot solve sudden traffic accidents and unconventional traffic conditions. Although the equipment used in the linear statistical model is relatively simple and low cost, the real-time performance is poor.

Nonlinear theoretical models to predict traffic flow by finding the original features of the traffic system in high-dimensional space through phase space reconstruction include wavelet analysis models (Ouyang et al., 2017), chaos theory models (Jieni and Zhongke, 2008), and mutation theory-based models. Nonlinear models have some advantages in the processing of time series, but there are disadvantages, such as more complex models and large computational effort.

The current mainstream models are neural networks, deep learning models, etc. (Yi et al., 2017), and the commonly used one is the BP neural network. Using deep learning long short-term memory neural networks (LSTM) (Kang and Zhang, 2020), MF-CNN (Yang et al., 2019), etc., traffic flow features are extracted from the temporal perspective for prediction. DMVST networks (Yao et al., 2018), ST-ResNet (Zhang et al., 2017), and traffic flow features are mined from the spatial perspective using convolutional neural networks (CNN). Currently, more and more researchers are studying the characteristics of traffic flow from both spatial and temporal perspectives to make more accurate predictions. For example, the ASTGCN model (Guo et al., 2019), an attention-based spatiotemporal graph convolutional network, consists of three independent components that model three temporal characteristics of traffic flow: current dependence, daily cycle dependence, and weekly cycle dependence. The STGCN model (Yu et al., 2017) is where STGCN effectively captures comprehensive spatiotemporal correlations by modeling multiscale traffic networks. The DCRNN model (Li et al., 2017) models traffic flow as a diffusion process on a directed graph and introduces a diffusion convolutional recurrent neural network (DCRNN) that captures spatial correlation using bidirectional random wandering on the graph, and captures temporal correlation using an encoder–decoder architecture with scheduled sampling.

Traditional convolutional networks, such as CNNs, have strong feature extraction and integration capabilities. CNNs are able to learn the pixel arrangement patterns in images by iterative updates of the convolutional kernel parameters to learn different shape features and spatial features. However, CNNs process data with very regular structured networks, i.e., very neatly arranged matrices, which are difficult to process for data with topological graph structure. The traffic flow data we study has a lot of irregular data structure, which requires the use of graph convolutional network to process, and the essence and purpose of the graph convolutional network is to mine the spatial features of the topological graph. In real life, there are many irregularly shaped data structures. Graph structures that are more typical, such as traffic road networks, social networks, chemical structures, and so on, do not have a regular internal structure like pictures or language. Graph structures are generally irregular; each node in the graph is unique around the structure, for this structure of data. With the use of traditional CNN, RNN network cannot be solved, or the effect is not ideal.

Inspired by the above research, this paper used both graph convolutional network (GCN) (Kipf and Welling, 2016) and gate recurrent unit (GRU) (Cho et al., 2014) to mine the spatiotemporal characteristics of traffic flow, and improved the GRU network by proposing the IM module, which enables a richer interactive representation between the input of the current moment and the hidden state passed down from the previous moment. This model is called the IMgru model, which enhances the significant information and weakens the secondary information, and enhances the modeling capability to better predict the traffic flow in the next moment.

In addition, we also found that the traffic flow at the four intersections in the Wenyi Road dataset has the same trend at the same moment, which is due to the interaction of traffic flow between upstream and downstream roads, indicating that the traffic flow is spatially correlated, so the IMgruGcn model was proposed, which combined the GCN module and the IMgru module to obtain the spatial and temporal characteristics of traffic flow, making the traffic flow prediction results more accurate. Comparing the GruGcn model with the IMgruGcn model proved the effectiveness of our proposed IM module, and comparing the IMgru model with the IMgruGcn model proved the effectiveness of combining the spatial module with the temporal module. According to the temporal and spatial correlation of the traffic flow, it is more effective to predict the traffic flow at the next moment.

## Data

**FIGURE 3:**
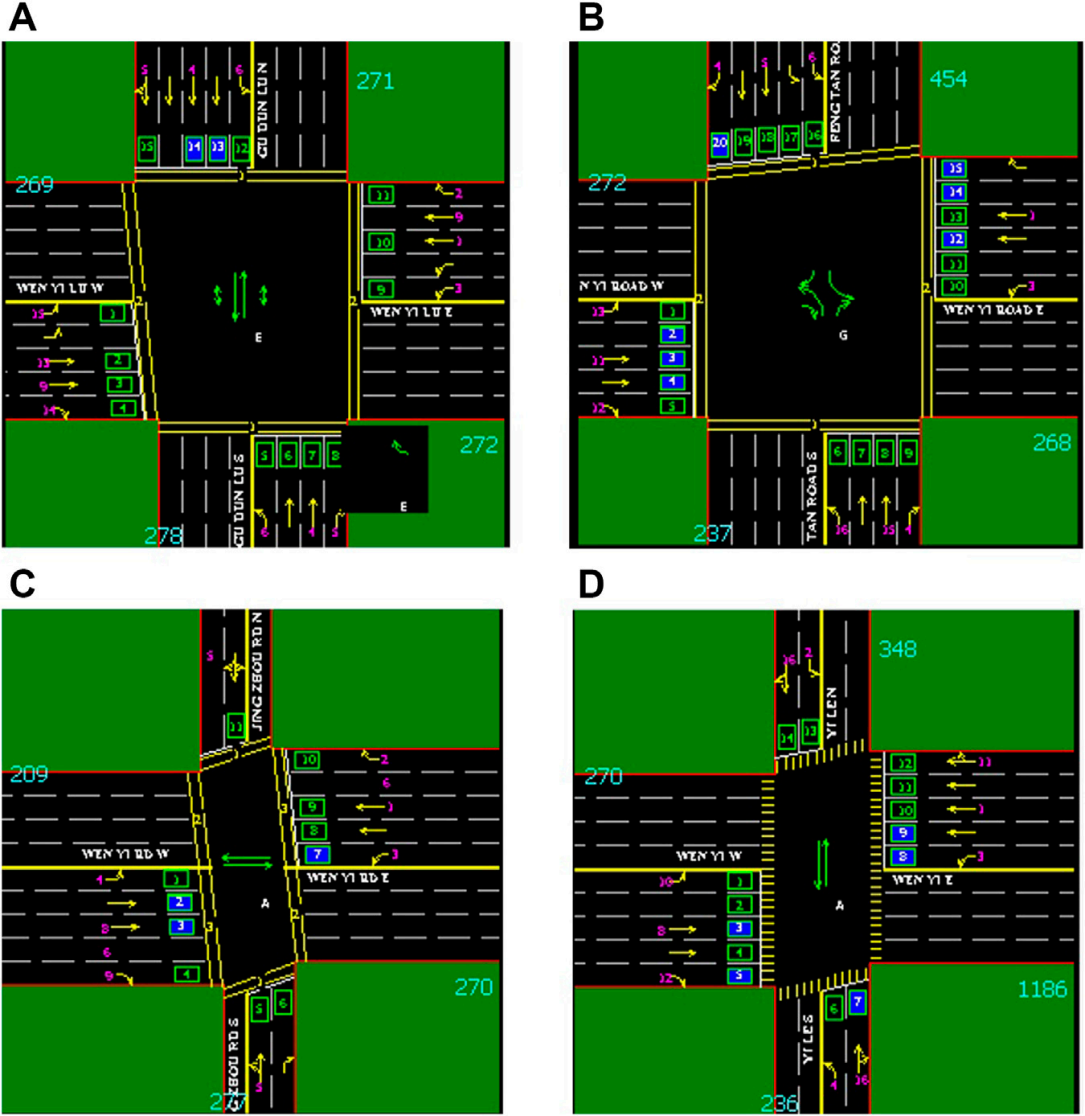
Channelization diagram of four intersections on Wenyi Road. **(A)** Channelization map of the Gudun intersection. **(B)** Channelization map of the Fengtan intersection. **(C)** Channelization map of the Jingzhou intersection.**(D)** Channelization map of the Yile intersection. Wenyi Road is an east–west road; there are four crossroads on this road: from west to east are Gudun Road, Fengtan Road, Yile Road, and Jingzhou Road.

The dataset for this experiment was collected from the traffic flow data of Wenyi Road in Hangzhou, Zhejiang Province. Figure 3 shows the channelization map of the four crossroads of Wenyi Road. The collection time was from August 1, 2020 to August 30, 2020. Detectors were placed at each of the four crossroads, and data were collected every 3 min. The collected data mainly include road code, lane code, collection time, and traffic volume. This experiment collected the time and the corresponding traffic volume of these two key data as the main content of the dataset. At the Gudun intersection, there are nine lanes on the north–south road 271 and road 278, and 10 lanes on the east–west road 269 and road 272. The sum of the number of vehicles passing in all these lanes at the same moment was taken as a sample. A sample was collected every 3 min. The same method was used in the other three intersections. A total of 480 × 4 samples were collected for 24 h per day. Due to a restarted sensor or some unpredictable errors, the value of traffic flow may be less than zero, and these data were removed. A total of 14,047 × 4 samples were collected in 30 days.

Vehicles are dense during peak periods and more susceptible to weather and traffic accidents, so the prediction is less effective. In order to predict off-peak traffic flow more effectively, this experiment divided the traffic flow into peak period and off-peak period for separate prediction. The traffic peak period in Hangzhou is divided into morning peak and evening peak. The morning peak time is 7:00–9:00, the evening peak time is 16:30–18:30, and the other time is recorded as off-peak time. A total of 80 samples were collected per day during the peak period. A total of 2,400 samples were collected in 30 days. During the off-peak period, a total of 11,660 samples were collected in 30 days. For each dataset, the first 80%, which is the first 3 weeks of traffic flow data, was selected as the training set, and the last 20%, which is the last week of traffic flow data, was used as the test set.

The adjacency matrix and feature matrix, constructed based on the Wenyi Road dataset, were used to represent the spatial relationship and temporal connection of roads, respectively. The adjacency matrix represents the spatial adjacency between the roads. Each row of the matrix represents a road, and there are four roads in the Wenyi Road dataset. So, the adjacency matrix dimension is 4 × 4. The matrix contains only two values of 0 and 1, and 1 means two roads are adjacent, and 0 means two roads are not adjacent. The feature matrix represents the number of vehicles passing on the four roads at different times. Each column is one road, and each row represents the traffic flow on the road at different time periods. The traffic flow was collected every 3 min, and the feature matrix dimension is 14,047 * 4. The value in the matrix indicates the traffic flow samples collected at different time periods, with a total of 14,047 * 4 samples.

The adjacency matrix and feature matrix were used as the input data of the model. The normalized feature matrix was input into the graph convolutional network to mine the spatial characteristics of traffic flow. Then a matrix of the same dimension was output as the input matrix of the IMGRU module, and the IMgru module extracted the temporal characteristics of traffic flow to predict the traffic flow at the next moment. The output matrix of the model was inverse normalized and compared with the real traffic flow data. Finally, the prediction capability of the model was calculated by evaluating the metrics.

## Methodology

### Temporal module

Traffic flow data are a time series, so they are considered to be solved by using recurrent neural network (RNN), which is effective for data with sequence characteristics and can mine the temporal information as well as semantic information in the data. However, the traditional RNN is prone to the gradient disappearance problem and cannot solve the long-term dependence problem. The Gated Recurrent Unit (GRU) (Cho et al., 2014) can solve this problem and has a simpler structure and faster operations. However, the inputs and of the traditional GRU model are independent of each other before they are input into the interior of the GRU model. They only interact with information inside the GRU to obtain the output of each gate, which may lead to the loss of valid information. Drawing on the application of LSTM model in natural language processing, MOGRIFIER LSTM, improves the generalization ability of language model (Melis et al., 2019). This paper proposes the IMgru model, which makes the input and the hidden state of the upper and lower moments have a richer interactive representation, enhances the significant information, weakens the secondary information, and enhances the modeling capability of the model.

**FIGURE 4:**
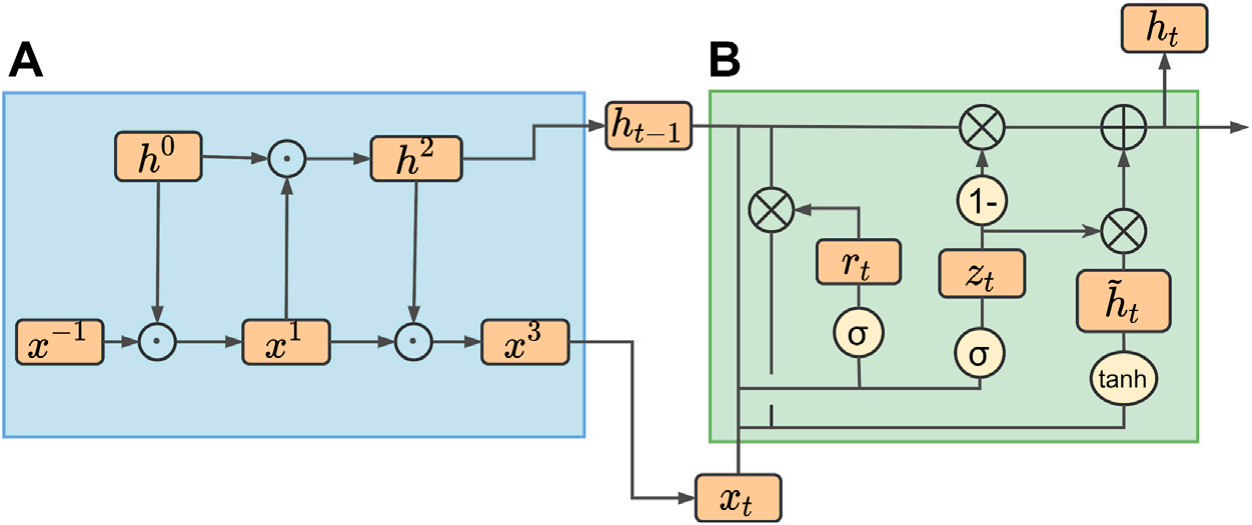
IMgru model structure. **(A)** IM module. **(B)** GRU module.

The structure of the conventional GRU model is shown in module B in Figure 4. $h_{t-1}$ denotes the hidden state at time t − 1, $x_{t}$ denotes the traffic information at time t, $r_{t}$ denotes the reset gate, $z_{t}$ denotes the update gate, and $h_{t}$ is the output state at time t. GRU takes the hidden state at time t − 1 and the current traffic information as input, and obtains the prediction result and hidden state at time t.

The reset gate decides how much of the information of the hidden state $h_{t-1}$ of the previous moment to reset, combining the new input with the previous memory. The update gate controls the hidden state $h_{t-1}$ of the previous moment, balancing it with the input information of the current moment, forgetting some information in $h_{t-1}$ passed down from the previous moment, and adding some information from the input of the current moment.

The overall process of the gating mechanism of GRU is as follows:

$$z_{t}=\sigma \left(W_{z}\cdot \left[h_{t-1},x_{t\;\;}\right]\right)$$ (1)

$$r_{t}=\sigma \left(W_{r}\cdot \left[h_{t-1},x_{t\;\;}\right]\right)$$ (2)

$${\tilde{h}}_{t}=tanh\left(W\cdot \left[r_{t}*h_{t-1},x_{t\;\;}\right]\right)$$ (3)

$$h_{t}=\left(1-z_{t}\right)*h_{t-1}+z_{t\;\;}*{\tilde{h}}_{t}$$ (4)

The input of the GRU model consists of two parts, the current input $x_{t}$ and the hidden state $h_{t-1}$ passed down from the previous node, which contains the information related to the previous node.

Theoretically, the current input should be related to the hidden state of the previous moment, but Figure 4 shows that the inputs $x_{t}$ and $h_{t-1}$ of the GRU model are independent of each other before they are input into the interior of the model. They only exchange information in the gating mechanism inside the GRU to obtain the output of each gate, which may lead to the loss of valid information allowing the current input to fully interact with the previous hidden state before inputting into the GRU may improve the results.

Due to the abovementioned shortcomings of the GRU model, we proposed an improvement method. The improved part is shown in Figure 4 module A. The improved model is called the IMgru model. By introducing additional gating operations, the current input $x_{t}$ and the hidden state $h_{t-1}$ passed down from the previous moment to be computed in multiple interactions before being input to the GRU. Eqs. 5 and 6 are their calculation methods.

$$x_{i}=2\sigma \left(Q^{i}h_{i-1}\right)⊙x_{i-2}\;\;\;\;\;\;\;\;\;\;\;\;\;\;\;\;\;\;\;for\;odd\;i\;\in \left[1\cdot \cdot \cdot r\right]$$ (5)

$$h_{i}=2\sigma \left(R^{i}x_{i-1}\right)⊙h_{i-2}\;\;\;\;\;\;\;\;\;\;\;\;\;\;\;\;\;\;\;\;for\;even\;i\;\in \left[1\cdot \cdot \cdot r\right]$$ (6)

where $x_{-1}=x,\;h_{0}=h$ , and r refers to the number of rounds of interaction, which controls how $x_{i}$ and $h_{i}$ should interact. When i is odd, Eq. 5 is operated, and when i is even, Eq. 6 is operated. When r=0 , the whole model becomes the original GRU network. Q and R are the weight matrices.

Each formula is multiplied by a constant 2 because after a sigmoid operation, the values are distributed in the interval (0, 1). By repeatedly multiplying, the value will become smaller and smaller, and finally reaches zero; therefore, multiplying by 2 ensures the stability of its value.

From Eq. 1 to Eq. 4, we know that the output hidden state $h_{t}$ at moment t is related to the input $x_{t}$ and the hidden state $h_{t-1}$ passed down from the t − moment. The hidden state calculated at the current moment is related to the input at the next moment. Thence, the hidden state passed down from the previous moment is also related to the input at the current moment.

However, the current moment input $x_{t}$ and the hidden state $h_{t-1}$ passed down from the previous moment are independent of each other before input into the GRU model. Just do a little calculation in the gating structure, which may lose some useful information. This paper decides to do some interaction operation on the input $x_{t}$ and the hidden state $h_{t-1}$ from the previous moment before the gating structure of the GRU model. The interaction way is shown in module A in Figure 4; the values of $x_{t}$ and $h_{t-1}$ are updated alternately by the value of i.

This improved approach allows a richer interactive representation of the input and the hidden state, enhancing the salient information, diminishing the secondary information, and enhancing the modeling capability of the model.

### Spatial module

**FIGURE 5:**
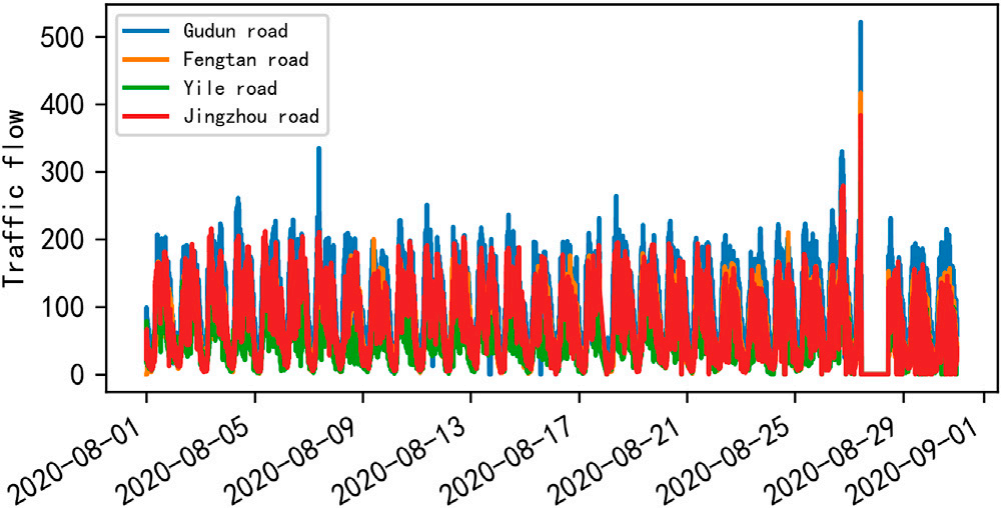
Mean visualization of the traffic volume of the four roads on Wenyi Road in 30 days.

After visualizing the traffic flow of the four roads in the Wenyi Road dataset for 30 days, as shown in Figure 5, it was found that there was a strong similarity in the changing trend of traffic flow of the four roads at the same time, which may be due to the spatiotemporal correlation of traffic flow, and the four roads affect each other spatially; thus, the traffic flow has similar changes at the same moment.

**FIGURE 6:**
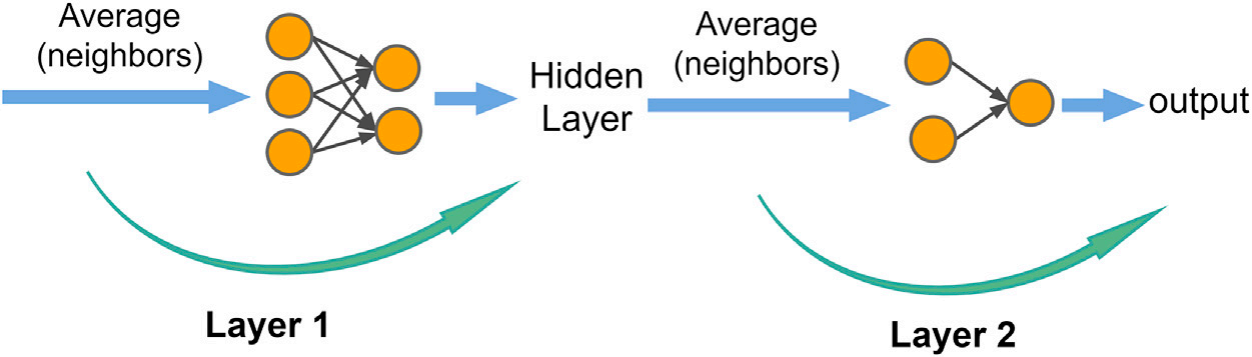
Graph convolutional network structure.

After discovering this spatial correlation of traffic flow, it is not accurate enough to rely on temporal correlation alone to predict traffic flow. In order to obtain more effective prediction results, the spatial module was combined with the temporal module to extract both spatial and temporal features of traffic flow. In order to obtain more effective prediction results, the spatial module (as shown in Figure 6) was combined with the temporal module to extract both spatial and temporal features of traffic flow (Zhao et al., 2019).

**FIGURE 7:**
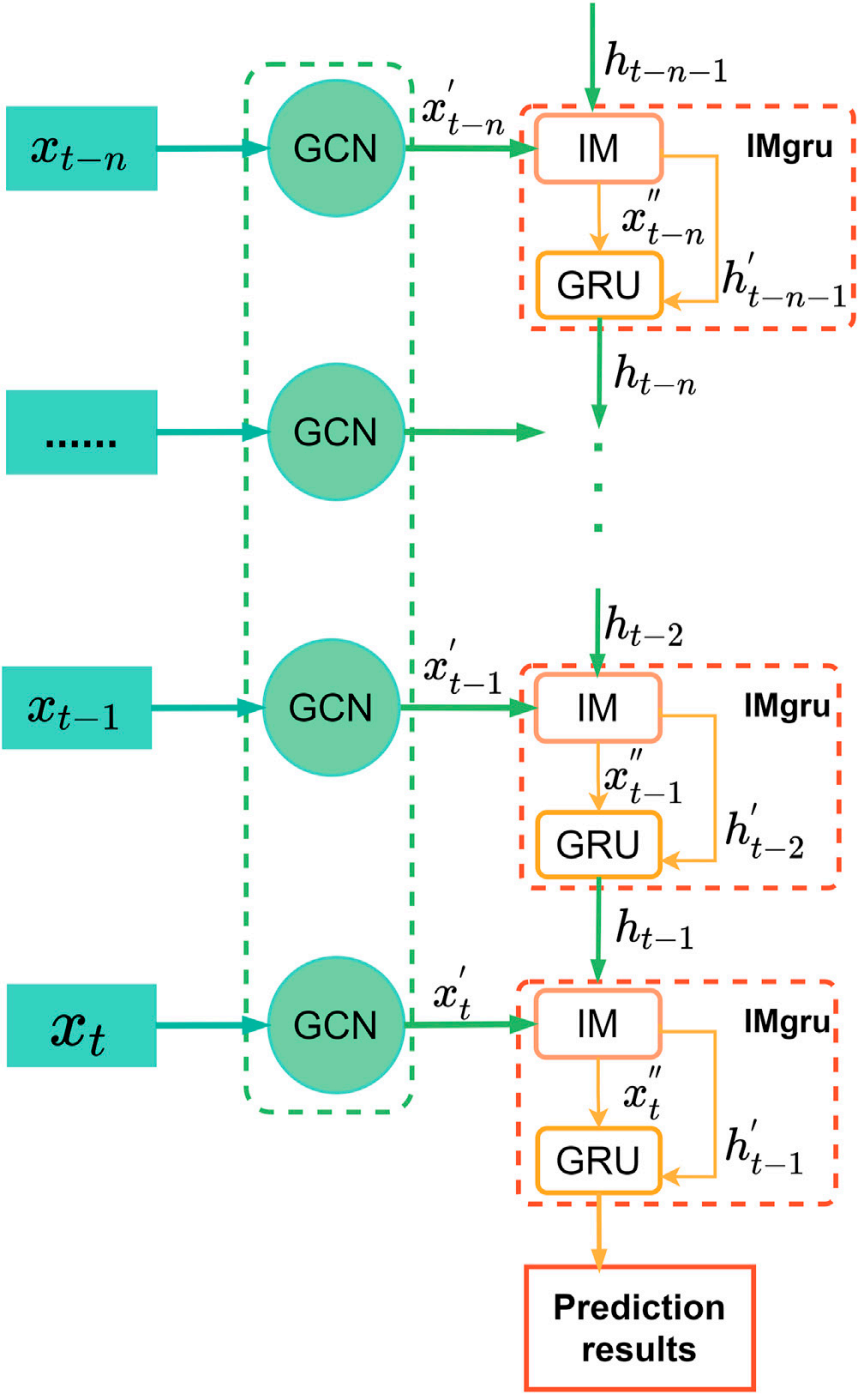
IMgruGcn model structure.

The spatiotemporal module was combined in the way shown in Figure 7, which combined graph convolutional networks (GCNs) and the IMgru model to extract the spatiotemporal features of the traffic flow simultaneously. Graph Convolutional Networks (Kipf and Welling, 2016) were used to extract the spatial features of the traffic flow, as shown in Figure 6. For each node, feature information of all its neighboring nodes was extracted, including the own features of the node. Then the average values obtained from these calculations are input to the neural network. Thus, the features of the graph structure data, which are the spatial features of the traffic flow, were extracted, and then the traffic flow data were input into the IMgru model to predict the traffic flow at the next moment based on the spatial and temporal correlation of the traffic flow.

There are two methods for extracting spatial features from graph convolutional networks: the spatial domain method and the frequency domain method (spectral method). The spatial domain method needs to find out the neighbors adjacent to each vertex, but the size and number of the neighbors of each vertex is inconsistent, so the computational processing must be specific to each node, which is more difficult to handle. The spectral method is to change the original graph from the node domain to the spectral domain and then define the convolution kernel in the spectral domain. Therefore, the graph convolution network in this paper uses the spectral method.

The traffic road network is abstracted as an undirected graph with N nodes in the graph. Each node has its own features. The features of these nodes are summarized as an N × D-dimensional feature matrix X (N is the number of nodes, D is the number of input features), and then the relationship between each node will also form an N × N-dimensional matrix called the adjacency matrix A. Feature matrix X and adjacency matrix A are the inputs to the model.

Each hidden layer in the graph convolution can be represented by a nonlinear function.

$$H^{\left(l+1\right)}=f\left(H^{\left(l\right)},A\right)$$ (7)

where the input layer $H^{\left(0\right)}=X$ , $H^{\left(l\right)}=Z$ , and l denotes the number of layers. The propagation between layers is given by Eq. 8.

$$f\left(H^{\left(l\right)},A\right)=\sigma \left(AH^{\left(l\right)}W^{\left(l\right)}\right)\;\;\;\;\;\;\;\;\;\;$$ (8)

where $W^{\left(l\right)}$ is the weight matrix of the *l*th layer, and σ is the activation function of the nonlinear regression. This experiment uses a two-layer graph convolution with the following procedure:

$$f\left(X,A\right)=\sigma \left(\tilde{A}\;relu\left(AXW_{0}\right)W_{1}\right)$$ (9)

where $W_{0}$ denotes the weight matrix from the input layer to the hidden layer, $W_{1}$ denotes the weight matrix from the hidden layer to the input layer, relu is the activation function, and $f\left(X,A\right)\in R^{N*T}$ represents the output of prediction time length of T. The multiplication of matrix A and matrix H in Eq. 8 indicates that the features of the neighboring nodes of each node are added to get the input of the next layer, but the features of its own nodes are not included. The features of the neighboring nodes are obtained, while ignoring the information of its own nodes. Hence, by adding the adjacency matrix with the identity matrix, $\tilde{A}=A+I$ is obtained, so that it includes the features of its own nodes. In general, the graph convolution operation is to obtain the weighted average of the features of each node and its neighbors, and then propagate them to the next layer to obtain spatial features. The process is shown in Figure 6.

After extracting the spatial characteristics of the traffic flow through GCN, the IMgru model is used to extract the temporal characteristics of the traffic flow. According to the temporal and spatial correlation of the traffic flow, the GCN module and the IMgru module are combined to predict the traffic flow in the next moment more effectively.

### Overall structure of the model

Figure 7 shows the total network structure. The input on the left side of the figure is the raw data of traffic flow, including the adjacency matrix and the feature matrix. Since the feature data are large, and the model converges slowly, the feature matrix needs to be linearly transformed from the input to the spatial feature extraction part, and the min–max normalization method is used to map the result to the interval (0, 1). The specific formula is as follows:

$$x=\frac{x-min}{max-min}$$ (10)

where min denotes the minimum value in the traffic flow data, and max denotes the maximum value in the traffic flow data. The normalized data are easier to handle and can speed up the convergence of the model.

The input $x_{t-\text{n}}$ at the current moment is processed by the graph convolution network, and the spatial features of the traffic flow are extracted. Then the traffic flow data with spatial features are input to the IMgru module. The IMgru module consists of the IM module and the GRU module. The data of $x_{t-n}^{'}$ link are the traffic flow data with spatial features obtained by GCN and will be input into the IMgru module. The top-to-bottom links in the IMgru module are the hidden state passed down from the previous moment of the GRU unit.

Since the first GRU unit does not have the hidden state passed down from the previous moment, the first hidden state is initialized, which is the first longitudinal link $h_{t-n-1}^{'}$ in the IMgru module, as a zero matrix with the same dimension as the feature matrix. After the current input and the hidden state passed from the previous moment are interacted by the IM module for multiple rounds, they are input to the GRU module to mine the time characteristics of the traffic flow.

By combining the GCN module and IMgru module to obtain both spatial and temporal characteristics of traffic flow, the IM module was proposed to mine temporal features, making the input and the hidden state of the upper and lower moments have a richer interactive representation, enhancing the significant information, diminishing the secondary information, and enhancing the modeling capability of the model to better predict the traffic flow in the next moment.

## Experiments and results

### Measurement indexes

This experiment uses the root mean square error (RMSE) and the mean absolute error (MAE) as the evaluation index of the model, which are commonly used to measure the accuracy of the variables. The calculation formula is:

$$RMSE=\sqrt{\frac{1}{m}\sum_{i=1}^{m}{\left(y_{i}-{\hat{y}}_{i}\right)}^{2}}$$ (11)

$$MAE=\frac{1}{m}\sum_{i=1}^{m}\left|y_{i}-{\hat{y}}_{i}\right|$$ (12)

where $y_{i}$ represents the true value of traffic flow, which is a sample collected, i.e., the sum of the number of vehicles passing in the four directions of east, west, north, and south at each intersection; ${\hat{y}}_{i}$ represents the predicted value of traffic flow; and m represents the number of observed traffic flow samples.

The root mean square error (RMSE), which can also be called standard error, measures the average size of the error. It is the square root of the mean of the squared deviation of the predicted value from the true value. The mean absolute error (MAE) is the average of the absolute errors of the predicted and true values. Therefore, the smaller the values of RMSE and MAE, the better.

### Environment settings

All experiments in this paper were programmed in the framework of the PyTorch environment, using the Python language and the PyCharm editor, and trained and tested on Intel(R) Core(TM) i5-7500 CPU @3.40 GHz processor, GPU: NVIDIA GeForce GTX 1070.

## Results and analysis

In order to compare the ability of the models proposed in this paper to predict traffic flow, this experiment was trained and tested on the peak period, off-peak period, and complete dataset of Wenyi Road. Each dataset consisted of adjacency matrix and feature matrix as input. The three models proposed in this paper, IMgru, GruGcn, and IMgruGcn, were compared with other traditional models including HA, SVR, ARIMA, GCN, and GRU for traffic flow prediction results. The model was also compared with the state-of-the-art method ASTGCN (Guo et al., 2019). The output was the predicted traffic flow matrix, which was compared with the feature matrix of the real traffic flow. The root mean square error (RMSE) and mean absolute error (MAE) were used as evaluation indexes to calculate the error between the predicted and real values. The prediction performance of each model was evaluated in Table 1 and Table 2. Table 1 compared the prediction performance of each model on the training set of Wenyi Road dataset, and Table 2 was the prediction performance on the test set of each dataset. From Table 1 and 2, it is obvious that the value of MAE is smaller than that of RMSE, which is because RMSE is the accumulation of the square of the error before opening the square, and it actually magnifies the gap between larger errors. The MAE, on the other hand, responds to the true error. Thus, the smaller the value of RMSE in the measurement, the greater its significance because its value reflects that its maximum error is also relatively small.

**TABLE 1:** The prediction results of the training set of peak period, off-peak period, and Wenyi Road complete dataset on each model.

Model	Off-peak		Peak		Complete dataset		Losloop		Shenzhen	
	Root mean square error (RMSE)	Mean absolute error (MAE)	RMSE	MAE	RMSE	MAE	RMSE	MAE	RMSE	MAE
HA	18.90	14.94	38.14	29.39	28.36	21.04	20.30	19.58	6.80	4.74
SVR	17.64	13.75	39.78	29.37	30.81	23.96	12.56	10.49	6.82	4.62
ARIMA	59.46	41.76	101.98	95.58	79.40	68.77	59.28	58.29	14.34	12.12
Graph convolutional network (GCN)	10.97	8.66	34.49	27.68	22.46	16.89	6.16	4.42	5.54	4.15
GRU	10.99	8.67	32.64	27.06	22.56	17.79	6.70	3.83	5.58	4.27
ASTGCN	8.91	6.67	**28.64**	**21.32**	19.11	15.07	10.95	6.72	7.68	5.14
IMgru (ours)	9.53	7.18	30.98	24.99	**18.66**	14.91	6.13	3.50	4.11	2.52
GruGcn (ours)	8.99	7.67	31.15	24.64	18.81	15.80	6.13	4.29	4.06	2.65
IMgruGcn (ours)	**8.29**	**6.47**	29.35	22.24	18.84	**14.26**	**5.52**	**2.77**	**3.62**	**2.46**

The bold values are represent the best prediction result of all models.

**TABLE 2:** The prediction results of the test set of peak period, off-peak period, and Wenyi Road complete dataset on each model.

Model	Off-peak		Peak		Complete dataset		Losloop		Shenzhen	
	RMSE	MAE	RMSE	MAE	RMSE	MAE	RMSE	MAE	RMSE	MAE
HA	20.42	15.74	49.04	42.11	42.75	30.86	20.02	19.07	6.98	5.71
SVR	19.76	15.41	47.45	40.52	40.06	28.61	13.09	11.03	8.26	6.53
ARIMA	54.36	37.65	103.83	98.21	90.11	78.98	58.94	57.87	13.67	11.56
GCN	14.11	10.49	40.68	33.76	29.19	20.13	8.65	6.08	6.01	4.48
GRU	14.21	10.45	39.99	32.86	27.46	20.95	8.84	5.96	5.64	3.26
ASTGCN	11.06	7.87	**35.02**	**28.83**	27.69	19.98	13.35	8.34	4.72	3.32
IMgru (ours)	11.83	8.50	38.69	32.05	26.54	20.28	5.64	3.36	4.20	2.92
GruGcn (ours)	12.19	9.45	39.22	32.96	27.21	20.73	7.42	5.16	4.27	2.98
IMgruGcn (ours)	**10.74**	**7.59**	38.12	31.88	**26.05**	**19.28**	**5.59**	**3.26**	**4.18**	**2.91**

The bold values are represent the best prediction result of all models.

The batch size was set to 32, which was too small to converge. Increasing the batch size will increase the processing speed, but the memory capacity will also increase.

The results in Table 1 and 2 all showed that our proposed models IMgru, GruGcn, and IMgruGcn improved the prediction results over other baseline networks, among which, the IMgruGcn model performed the best. The ARIMA model performed the worst due to its lack of spatiotemporal data processing capability, and the traditional statistical methods and machine learning methods had no effect of deep learning methods. On the peak dataset, the prediction effect of the ASTGCN model is better than the IMgruGcn model. However, the prediction effect of IMgruGcn model is better than the ASTGCN model on all the other four datasets.

**FIGURE 8:**
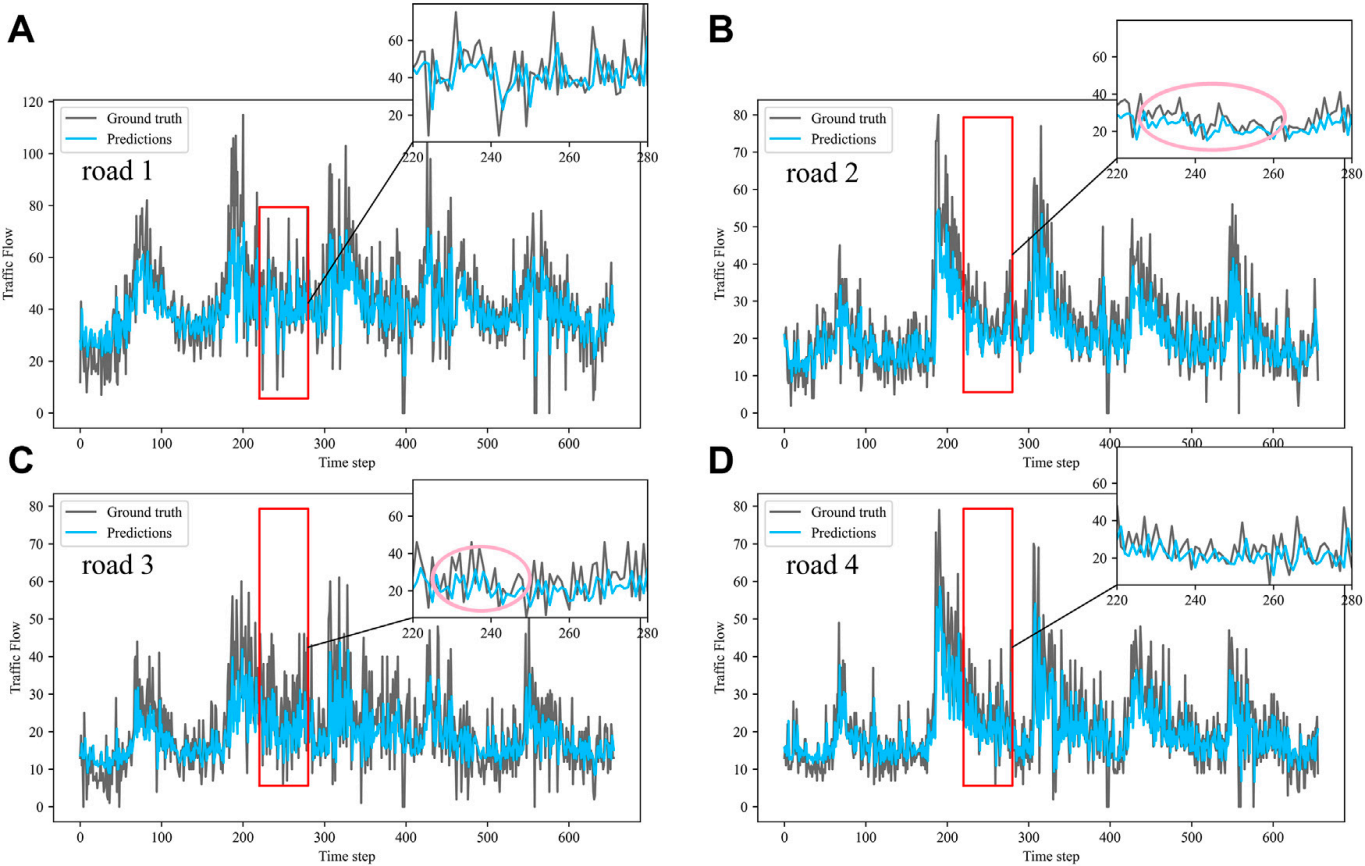
Visualization of IMgru model prediction results on off-peak dataset. **(A)** road 1 means Gudun Road. **(B)** road 2 means Fengtan Road. **(C)** road 3 means Yile Road. **(D)** road 4 means Jingzhou Road.

**FIGURE 9:**
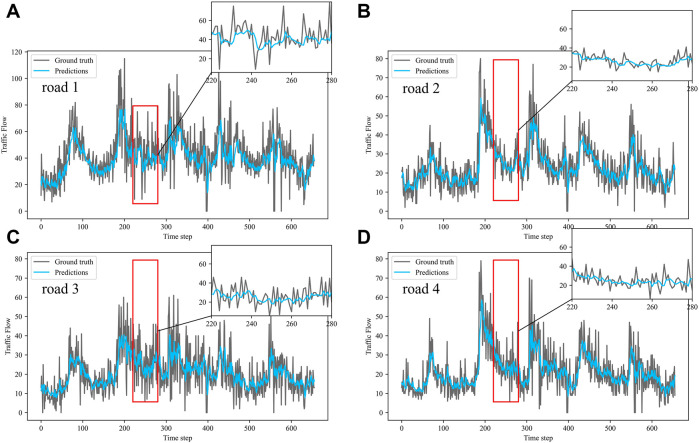
Visualization of GruGcn model prediction results on off-peak dataset. **(A)** road 1 means Gudun Road **(B)** road 2 means Fengtan Road. **(C)** road 3 means Yile Road. **(D)** road 4 means Jingzhou Road.

**FIGURE 10:**
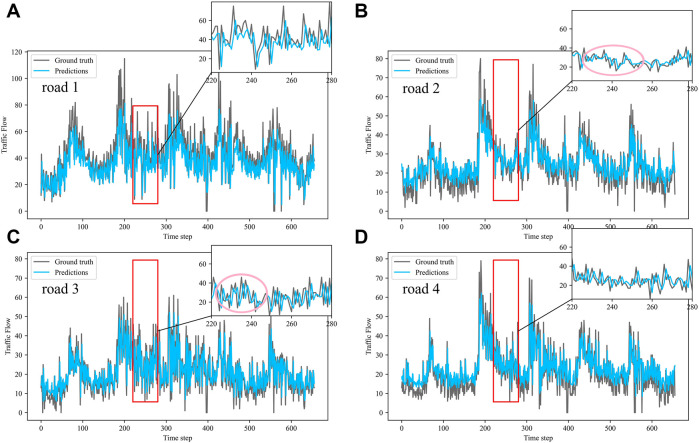
Visualization of IMgruGcn model prediction results on off-peak dataset. **(A)** road 1 means Gudun Road. **(B)** road 2 means Fengtan Road. **(C)** road 3 means Yile Road. **(D)** road 4 means Jingzhou Road.

**FIGURE 11:**
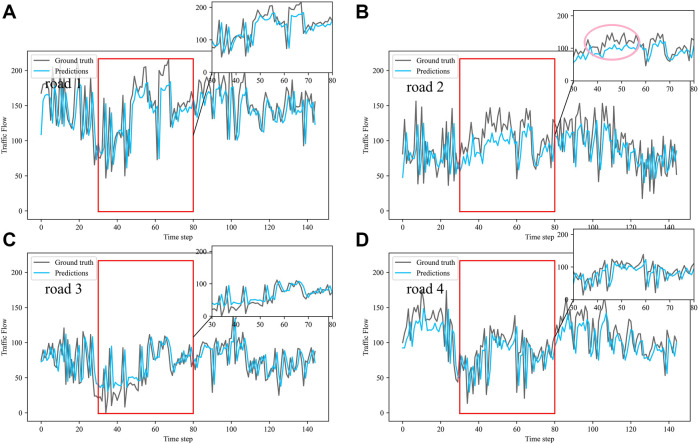
Visualization of IMgru model prediction results on peak dataset. **(A)** road 1 means Gudun Road. **(B)** road 2 means Fengtan Road. **(C)** road 3 means Yile Road. **(D)** road 4 means Jingzhou Road.

**FIGURE 12:**
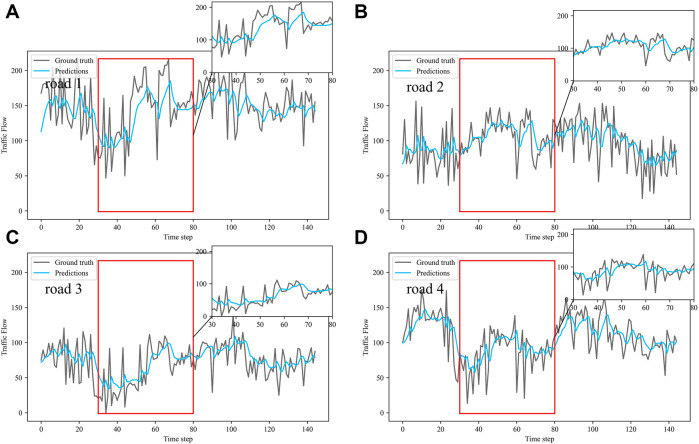
Visualization of GruGcn model prediction results on peak dataset. **(A)** road 1 means Gudun Road. **(B)** road 2 means Fengtan Road. **(C)** road 3 means Yile Road. **(D)** road 4 means Jingzhou Road.

**FIGURE 13:**
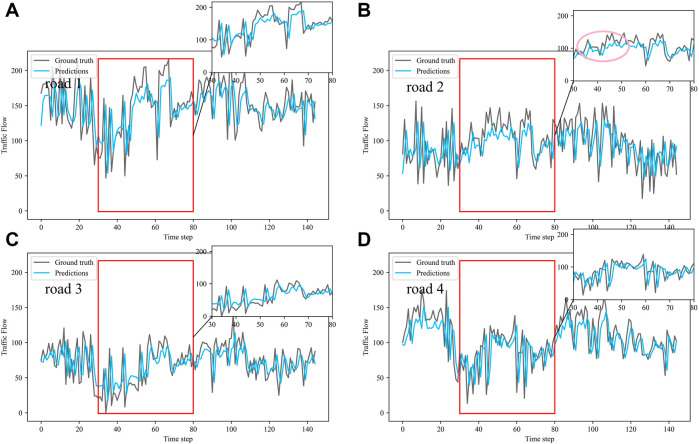
Visualization of IMgruGcn model prediction results on peak dataset. **(A)** road 1 means Gudun Road. **(B)** road 2 means Fengtan Road. **(C)** road 3 means Yile Road. **(D)** road 4 means Jingzhou Road.

**FIGURE 14:**
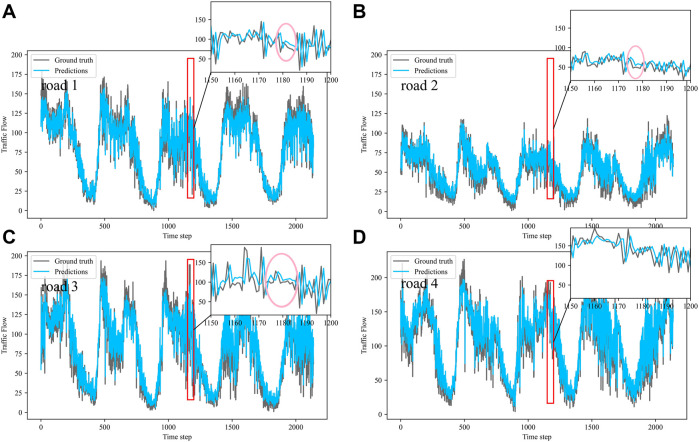
Visualization of IMgru model prediction results on the complete dataset. **(A)** road 1 means Gudun Road. **(B)** road 2 means Fengtan Road. **(C)** road 3 means Yile Road. **(D)** road 4 means Jingzhou Road.

**FIGURE 15:**
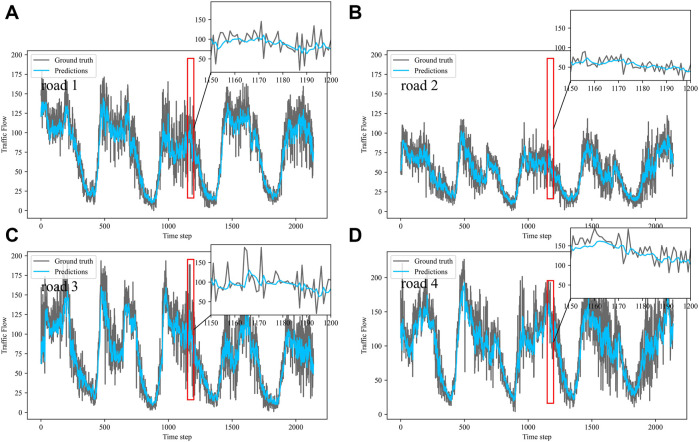
Visualization of GruGcn model prediction results on the complete dataset. **(A)** road 1 means Gudun Road. **(B)** road 2 means Fengtan Road. **(C)** road 3 means Yile Road. **(D)** road 4 means Jingzhou Road.

**FIGURE 16:**
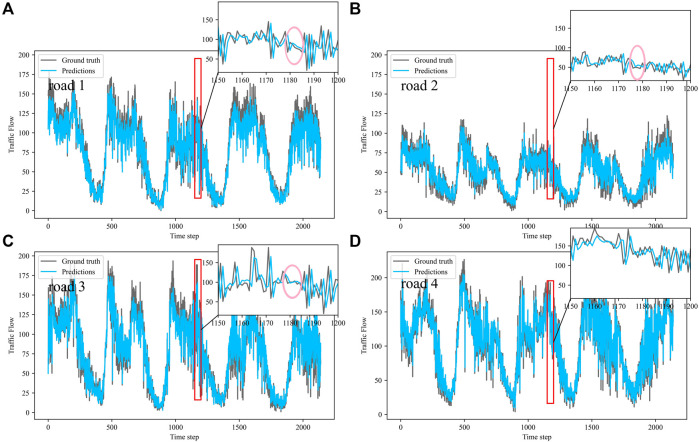
Visualization of IMgruGcn model prediction results on the complete dataset. **(A)** road 1 means Gudun Road. **(B)** road 2 means Fengtan Road. **(C)** road3 means Yile Road. **(D)** road 4 means Jingzhou Road.

Dividing the datasets into peak and off-peak periods to predict them separately, Table 1 and 2 showed that the prediction effect of the off-peak dataset was significantly better than that of the peak dataset. This was because the peak period was the time when people travel to work, and the traffic flow was intensive and vulnerable to weather and traffic accidents. The traffic flow during off-peak period was relatively stable, so the prediction effect was better. It can be seen that the traffic flow not only had spatial and temporal correlation but also was susceptible to external factors such as weather and traffic accidents. In order to see more clearly the traffic flow prediction effect of our proposed model, the prediction results of the three models, IMgru, GruGcn, and IMgruGcn, on the test set for four roads were visualized as shown in Figure 8, Figure 9, Figure 10, Figure 11, Figure 12, Figure 13, Figure 14, Figure 15, Figure 16

Figure 9 is a visualization of the prediction results of the GruGcn model. Obviously, the GruGcn model can only predict the general trend of traffic flow, and the predicted value was far from the real value. However, the prediction result of the IMgru model in Figure 8 is relatively better, which may be because road2 and road3 were less affected by the space of other roads. The spatial feature extraction of the graph convolutional network did not play a better role. In the visualizations of Figures 8 and 10, the pink ellipse in road1 and road3 showed that the prediction effect of the IMgru model was obviously not as well as that of IMgruGcn. It indicated the effectiveness of combining the IMgru model with the GCN model. Simultaneous extraction of spatiotemporal features of traffic flow is more effective than using temporal features alone to predict traffic flow.

From the visualization of the prediction results of the three models on the peak period, it can be seen that the prediction effect of the GruGcn model was much lower than that of IMgru and IMgruGcn. The GruGcn model can only predict the general trend of the traffic flow, and the prediction effect at the extreme values was poor. This showed the effectiveness of the IM module. The hidden state of the previous moment and the input of the current moment were made to interact continuously, enhancing the salient information and weakening the secondary information. The differences in the visualization also affected the results in Table 2. The value of RMSE for the GruGcn model was 0.5338 and 1.1077 higher than IMgru and IMgruGcn, and the value of MAE was 0.9037 and 1.0795 higher than IMgru and IMgruGcn. As can be seen in the pink ellipse of road2 in Figure 11, the IMgru model has slightly worse prediction results than the IMgruGcn model, which indicated the effectiveness of combining spatial module with the temporal module. The results in the visualizations above also affected the values in Table 2. The RMSE value and MAE value of the IMgru model were 0.5739 and 0.1758, respectively, higher than those of the IMgruGcn model.

In the visualization of the prediction results for the complete dataset, zoom in on the part of the region near the peak. The prediction results of the GruGcn model in Figure 15 show a smooth trend with no obvious extreme values. In contrast, the IMgru model with the IM module, as shown in Figure 14, had a more pronounced prediction at the extreme values. This gap in the visualization affected the values of the evaluation indicators in Table 2. The values of RMSE and MAE of the IMgru model were lower than those of the GruGcn model by 0.6699 and 0.4455. In the pink ellipse of road1, road2, and road3 in Figure 14, the difference between the predicted value and the true value was larger than in the IMgruGcn model. This also made the RMSE value and MAE value of IMgruGcn model lower than IMgru by 0.4907 and 1.0039. It indicated that extracting the spatiotemporal characteristics of traffic flow can be more effective for prediction.

Figures 6–8 show the visualization results of the true values and predicted values for the four roads in Wenyi Road. The horizontal axis is the time step, and the vertical axis is the traffic flow, showing the difference between the predicted and true values. They were tested separately using the peak period dataset, the off-peak dataset, and the complete dataset. In this way, the prediction performance of the three models, IMgru, GruGcn, and IMgruGcn, was compared. According to the results in Table 1, Table 2 and from the visualizations, the IMgruGcn model performed better than the other networks on the three datasets. It proved the effectiveness of our proposed IM module and the effectiveness of combining the spatiotemporal module to predict traffic flows. In addition, the off-peak dataset predicted better than the other datasets, which also proved the necessity of dividing the dataset into peak and off-peak periods for prediction separately.

## Discussion

Due to the increasing real-time nature of the data, the change pattern is also becoming less obvious and subject to stronger disturbances. Short-time traffic flow prediction is more influenced by random factors, more time varying and uncertain, and the difficulty of prediction is increasing. We use neural network models GRU and GCN, which can establish good input and output mapping models as long as there are large numbers of input and output samples, which are automatically adjusted by neural networks. It can guarantee the reliability of the prediction results, and has the characteristics of strong fault tolerance and robustness.

By visualizing the traffic flow of the four roads in Wenyi Road, it was found that the four roads had strong similarity in the changing trends of traffic flow at the same moment. This indicated that the traffic flow of the four intersections was spatially influenced by each other. It is not accurate to predict traffic flow only by the temporal characteristics of traffic flow. Therefore, the spatial characteristics of the traffic flow were added for more accurate prediction.

In putting the spatial feature module after the temporal feature module, the prediction performance became worse instead. It might be caused by the influence among the four crossroads. The traffic flow may change at different moments because of the spatial relationship of the crossroads. For example, at time t, the increase in the number of vehicles on Gudun road may lead to an increase in vehicles on Fengtan road at moment t + 1. Thus, the spatial features should be considered first and then the temporal features, to capture the spatiotemporal characteristics of the traffic flow, and when the spatial module was applied before the temporal module, a better prediction result was obtained, which indicated that there is a correlation between spatial and temporal, and the correlation can predict more effectively.

In predicting the temporal characteristics of traffic flow, first consider the use of the recurrent neural network, which passes the data from different moments into the recurrent neural network sequentially predicting the next moment by remembering the information of the previous moment. However, the input information that is too far apart is difficult to memorize. Thus, it cannot solve the long-term dependence problem, and may produce gradient disappearance or explosion problem. The long-term dependency problem can be solved by using variants of RNN networks, LSTM and GRU. Since the GRU model has a simpler structure, less computation, and can reduce the risk of overfitting, we chose to use the GRU model to obtain the temporal characteristics of the traffic flow.

For the traditional GRU model, the input at the current moment and the hidden state passed down from the previous moment are independent of each other until they enter the model interior. They only interact with information inside the GRU. This may lead to the loss of valid information. Therefore, some interaction operations were done on $x_{t}$ and $h_{t-1}$ before the gating structure of the GRU model, to make a richer interaction representation between the input and the hidden state, to enhance the modeling capability.

When combining spatiotemporal networks, convolutional neural networks (CNNs) were first considered to extract spatial features. It was found that CNN was not effective in extracting spatial features of traffic flow, which was because the pixel points in the image or video data processed by CNN were arranged into a very neat matrix with regular internal structure. Since the traffic road network was intricate, and each road has a unique surrounding structure, the traffic road network can be abstracted into a graph structure with irregular shape. With such structured data, it would be difficult to select a fixed convolutional kernel to accommodate the irregularity of the whole graph using traditional CNNs, such as the uncertainty of the number and the uncertain order of neighboring nodes. Graph convolutional networks (GCNs) can apply the convolutional neural networks used in deep learning for images to graph data and can solve the spatial structures with irregular shapes that cannot be handled by CNN networks. So this paper used GCN to extract the spatial features of traffic flow. Based on the spatiotemporal correlation characteristics of traffic flow, the GCN and the IMgru model were combined to extract the spatial and temporal characteristics of traffic flow for better prediction.

The model was experimented on a public dataset. The results showed that the proposed model outperformed the other models on the public dataset, which indicated the better generalization ability of our proposed model.

## Conclusion

To alleviate traffic congestion and facilitate drivers to choose roads reasonably, this paper proposed an effective traffic flow prediction method, which combined GCN with an IMgru model to extract the spatial and temporal features of traffic flow, respectively.

In this paper, the traffic flow dataset of four intersections of Wenyi Road in Hangzhou was collected. Due to the existence of rush hour in Hangzhou on weekdays, the traffic was very congested during this period. Traffic flow was highly susceptible to external factors such as weather and traffic accidents. Therefore, the prediction of traffic flow during peak hours was not very satisfactory. The dataset was divided into peak period and off-peak period to predict separately. It was expected that the off-peak dataset will have better prediction results. Experiments showed that the off-peak dataset had significantly better predictions than the peak dataset, and in the prediction effect of the complete dataset between the peak period and the off-peak period dataset.

Since traffic flow is a time-series data, the gated recurrent unit (GRU) was used to predict the traffic flow at the next moment by remembering the information of the previous period, keeping the valid information and discarding the useless information through the gating mechanism. Before the traffic flow data was input to the GRU model, this method made an improvement by doing some interaction operations between the input $x_{t}$ and the hidden state $h_{t-1}$ passed down from the previous time, so that the input and the hidden state had a richer interaction representation and prevented the loss of significant information. Then the features of the traffic flow information of the previous time were extracted, and the traffic flow of the next time period was predicted effectively.

The traffic flow of four crossroads in the Wenyi Road dataset had strong similarity changing trends at the same time, as shown in Figure 6, which might be due to the spatiotemporal correlation of traffic flow. The interdependencies were caused by the traffic flow effect between upstream and downstream roads. Thus, the traffic road network was abstracted as an undirected graph in the paper, and the features of the graph structure data were extracted by graph convolutional network (GCN). Then the spatial features were input into the IMgru model. The spatial and temporal characteristics of the traffic flow were extracted separately to predict the traffic flow at the next moment. The strategy not only improved the temporal feature extraction ability of the model but also mined the temporal–spatial characteristics of traffic flow.

In the training set of the off-peak dataset, the RMSE and MAE of the IMgru model were 1.4566 and 1.4838, respectively, lower than those of the GRU model. In the test set of the off-peak dataset, the RMSE and MAE of the IMgru model were 2.3678 and 1.9561, respectively, lower than those of the GRU model. This illustrated the effectiveness of our proposed IM module.

On the peak dataset, the prediction effect of the ASTGCN model is better than that of the IMgruGcn model. This may be due to the dense vehicles during the peak period and the more serious time variation and uncertainty of traffic flow. The ASTGCN model is able to capture this characteristic of the traffic flow better. However, the prediction effect of IMgruGcn model is better than the ASTGCN model on all the other four datasets. This indicates that the traffic flow prediction performance of IMgruGcn model is stronger.

In the training set of the off-peak dataset, the RMSE and MAE of the IMGruGcn model were 1.2417 and 0.7194, respectively, lower than those of the IMgru model. In the test set of the off-peak dataset, the RMSE and MAE of the IMGruGcn model were 1.0923 and 0.902, respectively, lower than those of the IMgru model. The effectiveness of combining spatiotemporal modules to extract spatiotemporal features of traffic flow was illustrated. IMgruGcn is a model that combines the IMgru module and the GCN module, which can effectively extract the spatiotemporal characteristics of traffic flow. The experimental results showed that the IMgruGcn model has the best prediction results on the four datasets.

In the future, external factors such as weather and traffic accidents are considered to be added to the prediction model to improve the prediction capability, and the network structure is optimized to achieve real-time prediction capability for better performance.

## Data Availability

The datasets presented in this article are not readily available because the data was collected through the local transportation department, not a public dataset. Requests to access the datasets should be directed to Xing Xu, xuxing@zust.edu.cn.
